# Role of the RAGE Axis during the Immune Response after Severe Trauma: A Prospective Pilot Study

**DOI:** 10.1155/2015/691491

**Published:** 2015-12-31

**Authors:** Florian Uhle, Christoph Lichtenstern, Thorsten Brenner, Thomas Fleming, Christian Koch, Andreas Hecker, Christian Heiss, Peter Paul Nawroth, Stefan Hofer, Markus Alexander Weigand, Katja Weismüller

**Affiliations:** ^1^Department of Anesthesiology, Heidelberg University Hospital, 69120 Heidelberg, Germany; ^2^Department of Medicine I and Clinical Chemistry, Heidelberg University Hospital, 69120 Heidelberg, Germany; ^3^Department of Anaesthesiology and Intensive Care Medicine, Justus-Liebig-University, 35392 Giessen, Germany; ^4^Department of General and Thoracic Surgery, Justus-Liebig-University, 35392 Giessen, Germany; ^5^Department of Trauma, Hand and Reconstructive Surgery, University Hospital of Giessen-Marburg GmbH, Campus Giessen, 35392 Giessen, Germany

## Abstract

*Background*. Severe traumatization induces a complex pathophysiology, driven by the patient's own immune system. The initial activation is a result of damage-associated molecular patterns, which are released from disrupted and dying cells and recognized by immune receptors, for example, RAGE. In this study we aimed to evaluate the contribution of the RAGE axis to early and late immune responses.* Methods*. We enrolled 16 patients with severe trauma together with 10 patients after major abdominal surgery and 10 healthy volunteers. Blood samples were taken on admission and every 48 h for a total of 8 days. Plasma concentrations of various RAGE ligands as well as RAGE isoforms and IL-6 were measured by ELISA. Monocyte surface expression of RAGE and HLA-DR was assessed by flow cytometry.* Results*. High and transient levels of IL-6 and methylglyoxal characterize the early immune response after trauma, whereas samples from later time points provide evidence for a secondary release of RAGE ligands.* Conclusion*. Our results provide evidence for a persisting activation of the RAGE axis while classical mediators like IL-6 disappear early. Considering the immunocompromised phenotype of the monocytes, the RAGE ligands might be substantial contributors to the well-known secondary stage of impaired immune responsiveness in trauma patients.

## 1. Introduction

The treatment of traumatic injuries is a basic everyday task of the healthcare systems around the world. The situation becomes drastically more challenging when the severity and combination of injuries result in a life-threatening condition for the patient. These severe traumatic injuries cause globally more than 5 million deaths per year and this number might further increase over the next years [1, 2]. Besides the actual fatality rate, the treatment, remaining disabilities, and bodily impairments represent a high individual as well as socioeconomic burden [3]. Severe injuries originate from a variety of causes, not least from armed conflicts, but in the absence of war, traffic accidents are the leading cause of hospitalization in developed as well as third-world countries. As a consequence of the injuries, the patient's homeostasis collapses and a highly complex pathophysiological network is triggered [4]. Main drivers at it are both interwoven systems of coagulation and inflammation [5], the latter one initially activated by immunogenic* damage-associated molecular patterns* (DAMPs) released from disrupted and necrotic cells [6]. Due to the large amount of injuries and released mediators, trauma patients often present with a systemic inflammatory response syndrome (SIRS) at first. This is a result of DAMPs activating immune cells by binding to their corresponding pattern recognition receptor, for example, the* Receptor for Advanced Glycation End Products* (RAGE). RAGE is a multiligand immune receptor that recognizes different DAMPs, including proteinergic ligands like* high-mobility group box 1 protein* (HMGB1) or S100 proteins as well as metabolically modified proteins and lipids, known as* Advanced Glycation End Products* (AGEs) [7–9]. As a consequence of RAGE binding, proinflammatory signaling cascades are activated, ultimately leading to the activation of NF-*κ*B and changes in target gene expression. AGEs thereby arise from a nonenzymatically reaction (Maillard reaction) in conditions of high oxidative stress in combination with the availability of glucose [10]. Several conditions, for example, diabetes and its complications like atherosclerosis, have been shown to be causally driven by these compounds and ligands [11] and soluble forms of RAGE have been identified and characterized in many others, including acute systemic inflammatory conditions like sepsis [12–14]. Two prominent soluble isoforms with distinct mechanisms of generation are known, endogenous secretory RAGE (esRAGE) and soluble RAGE (sRAGE). While esRAGE is the consequence of an alternative splicing event followed by secretion, sRAGE derives from the proteolytic cleavage of the membrane-bound receptor. After an initial activation, the organism tries to balance the immune reaction, leading to a compensatory phase of immune dysfunction in trauma patients [15]. This later phase is of high relevance to the patients, as they are rendered highly vulnerable for infections during this time of recovery, not only because of their immune status, but also due to the disruption of physical body barriers like, for example, skin or lung epithelium.

Following our earlier own results and results from other groups [16, 17], we wanted to extend our knowledge about the participation of RAGE and its ligands in the systemic pro- and anti-inflammatory immune response after trauma. In order to achieve this, we conducted a prospective observational cohort study and measured plasmatic concentrations of known ligands as well as soluble and membrane-bound receptor isoforms. Surprisingly, we are able to show different spatiotemporal patterns for the ligands of RAGE, hinting at a persistent basal “RAGEing” milieu, which might contribute to the immune dysfunction of the patients.

## 2. Patients and Methods

### 2.1. Patients

This observational clinical study was conducted in the Surgical Intensive Care Unit of the University Hospital Giessen, Germany, after approval by the local ethics committee (Ethics Committee of the Medical Faculty of the Justus Liebig University Giessen: Trial code 127/09/German Clinical Trials Register: DRKS00000480). Informed consent was obtained from all patients and volunteers enrolled. In case of a patient not being able to give consent, the legal representative was informed in advance. Overall, 16 patients after severe trauma as characterized by an Injury Severity Score (ISS) ≥ 16 were enrolled in this study. In addition, 10 patients after major abdominal surgery (Table 1) and 10 healthy volunteers were included. For all groups, the fulfillment of any of the following criteria led to an exclusion from the study: age < 18 years, terminal renal insufficiency, diabetes mellitus (types I and II), autoimmune disorder (e.g., Morbus Crohn, rheumatoid arthritis), atherosclerosis with history of myocardial ischemia/coronary artery bypass grafting/percutaneous transluminal coronary angioplasty, and pregnancy.

Blood samples were taken from trauma patients within 24 h after hospital admission and subsequently every 2 days (for a maximum of 8 days), as long as the patients were present in the ICU. Blood was drawn only once in case of surgical patients (within 24 h after end of surgery) and healthy volunteers.

### 2.2. Sample Preparation and ELISA

Blood was drawn directly into anticoagulative EDTA tubes and immediately processed. Plasmatic and cellular fractions were separated by centrifugation with 1.200 ×g for 10 minutes at room temperature. After removal, plasma was stored in aliquots at −80°C until further analysis. Every sample was only thawed once before analysis.

Plasmatic factors were analyzed using commercially available ELISA according to the manufacturer's instructions: sRAGE (R&D Systems, Minneapolis, USA), esRAGE (B-Bridge International, Cupertino, USA), interleukin-6 (IL-6) (R&D Systems, Minneapolis, USA), AGE, CML, and MG (all the three from Cell Biolabs, Inc., San Diego, USA), S100A8 (RayBiotech, Norcross, USA), S100A12 (MBL International, Woburn, USA), and HMGB1 (Shino-Test Corporation, Tokyo, Japan).

### 2.3. Flow Cytometry

Expression of RAGE and HLA-DR was assessed by flow cytometry. For the quantitative measurement of HLA-DR, 50 *μ*L of whole blood was incubated with 20 *μ*L of Quantibrite HLA-DR/monocyte antibody cocktail (BD Bioscience, San Jose, USA) for 30 minutes at 4°C in the dark. Afterwards, erythrocytes were lysed by the addition of 450 *μ*L 1x FACS lysing solution (BD Bioscience, San Jose, USA) and further incubation for 15 minutes at room temperature. Measurements were performed on a FACSCalibur cytometer (BD Bioscience, San Jose, USA). At each time point a calibration curve was obtained by measuring beads with distinct fluorescence properties (Quantibrite PE Beads, BD Bioscience, San Jose, USA). Results were calculated according to the manufacturer's instructions and given as average number of HLA-DR molecules on each monocyte.

In case of RAGE, 100 *μ*L whole blood was incubated for 30 minutes with 0.2 *μ*g anti-CD14-FITC (category number 301804, BioLegend, San Diego, USA) together with 4 *μ*g anti-RAGE-APC (category number sc-80652, Santa Cruz Biotech, Dallas, USA) or an equivalent amount of isotype control antibody (sc-2889, Santa Cruz Biotech, Dallas, USA). Erythrocyte lysis was performed by addition of 2 mL FACS lysing solution followed by an additional incubation (15 minutes) and two washing steps. Measured mean fluorescence intensity was compared to an APC calibration curve (Bangs Laboratories, Fishers, USA) to obtain a robust quantitative result expressed in molecules of equivalent soluble fluorochrome (MESF).

### 2.4. Measurement of Methylglyoxal with High-Performance Liquid Chromatography (HPLC)

The concentrations of methylglyoxal (MG) in plasma were determined by derivatization with 1,2-diamino-4,5-dimethoxybenzene and HPLC of the quinoxaline adduct by fluorescence detection as described earlier [18, 19].

### 2.5. Statistics

All analysis and visualizations were performed using SPSS Statistics for Mac version 21 (IBM, Armonk, NY). In general, boxplots were used for graphical representation. For group comparisons, the nonparametrical Mann-Whitney *U* test was used. For the comparison of more than two groups, Kruskal-Wallis test for global differences was performed initially. For analysis of correlation, nonparametric Spearman *ρ* analysis was performed. Levels of significance are depicted as symbols within the figures with ^*∗*^ *P* < 0.05, ^*∗∗*^ *P* < 0.01, and ^*∗∗∗*^ *P* < 0.001.

## 3. Results

### 3.1. Study Population

The investigated cohort of trauma patients mainly consisted of males (87.5%) of middle age (median: 44.1 years) with a median ISS of 34.1, representing a “typical” trauma cohort with severe injuries. These mainly resulted from traffic accidents, from which car accidents as a (co)driver were the leading cause (43.8%). As a consequence, the majority of patients presented with injuries of the thorax (87.5%) and/or the abdomen (75%), whereas head injuries were only present in around one-third of all patients (Table 1). In terms of routine laboratory markers, the group of trauma patients showed an early increase in CRP, while leucocytes had an overall delayed kinetic. Blood glucose levels tended to be increased over the whole observation time (Supplementary Figure  1) (see Supplementary Material available online at http://dx.doi.org/10.1155/2015/691491). The postoperative control cohort consisted of patients who were subjected to major abdominal surgery, for example, resection of the pancreas or stomach.

### 3.2. Soluble Isoforms of RAGE and IL-6 after Trauma

**Figure 1:**
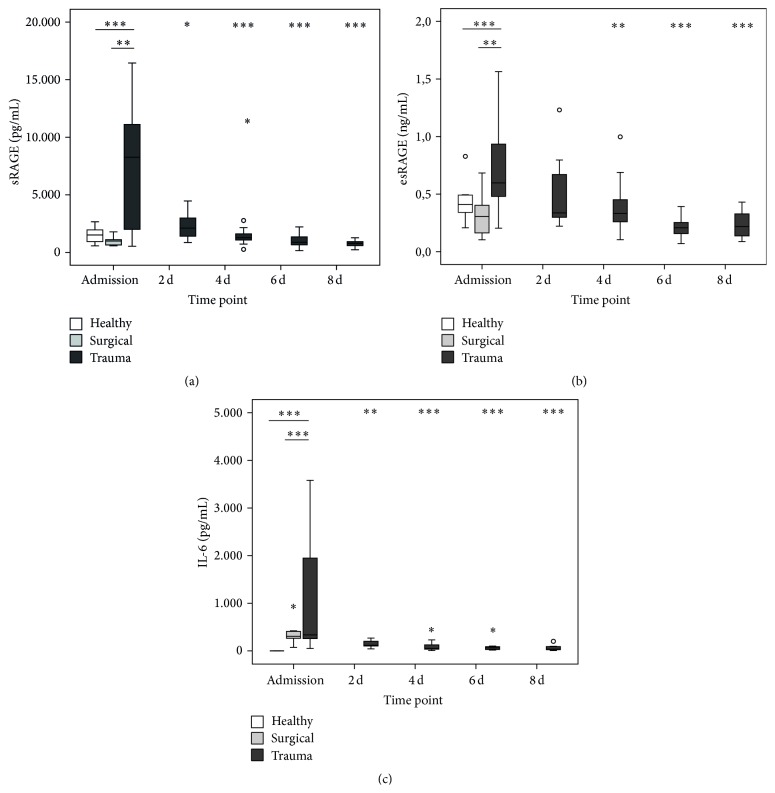
Plasma concentrations of RAGE isoforms and IL-6 after trauma. sRAGE (a), esRAGE (b), and IL-6 (c) levels at admission (*n* = 16/10/10 for trauma/surgical/healthy) and 2 d (*n* = 16), 4 d (*n* = 15), 6 d (*n* = 11), and 8 d after trauma (*n* = 11). ^*∗*^ *P* < 0.05, ^*∗∗*^ *P* < 0.01, and ^*∗∗∗*^ *P* < 0.001 compared to trauma at admission (Mann-Whitney *U*).

In line with our previous study, we can show an early and transient increase in both soluble isoforms of RAGE, sRAGE and esRAGE, and IL-6 immediately after trauma (Figure 1). Interestingly, these increased plasma levels cannot be observed after surgery, hinting at a major influence of a globally disturbed hemostasis as present in trauma patients compared to elective patients under general anesthesia.

### 3.3. Cell Surface Expression of RAGE and HLA-DR on Monocytes

**Figure 2:**
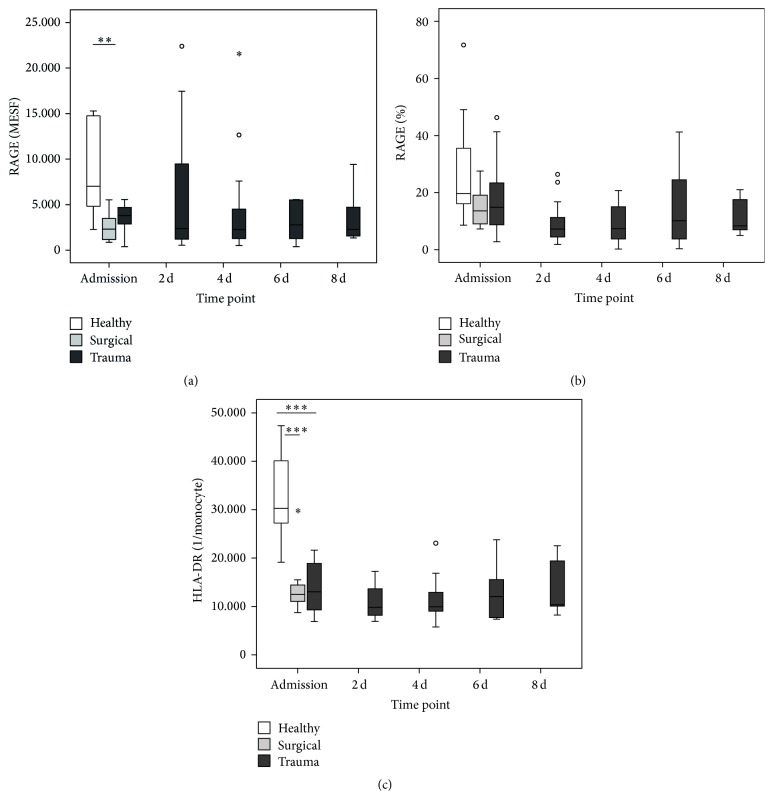
Cell surface expression of RAGE and HLA-DR. Results for RAGE are given as Molecules of Soluble Fluorochrome (MESF) (a) and percentage of RAGE^+^ monocytes compared to isotype (b), HLA-DR as mean amount of molecules per monocyte (c) at admission (*n* = 16/10/10 for trauma/surgical/healthy) and 2 d (*n* = 16), 4 d (*n* = 14 for RAGE), 6 d (*n* = 10), and 8 d after trauma (*n* = 8). ^*∗*^ *P* < 0.05, ^*∗∗*^ *P* < 0.01, and ^*∗∗∗*^ *P* < 0.001 compared to trauma at admission (Mann-Whitney *U*).

In order to assess the abundance of RAGE on monocytes, we performed flow cytometry measurements. Both patients with trauma and surgical patients showed an* ab initio* decreased abundance of RAGE on the cell surface (Figure 2(a)) as well as a trend to a general decrease of RAGE-positive monocytes compared to healthy controls (Figure 2(b)). These tendencies are maintained over all time points. In addition, as a surrogate marker of immune competence, we quantitatively measured monocytic HLA-DR surface expression. As expected, again both groups showed a significant decrease of HLA-DR expression, which persisted in the patients after trauma over the whole observation time. Despite the same overall kinetic, the expression levels of RAGE and HLA-DR in trauma patients over all time points correlate only weakly but yet significantly (*ρ* = 0.336, *P* = 0.007).

### 3.4. RAGE Ligands and Metabolic Stress after Trauma

**Figure 3:**
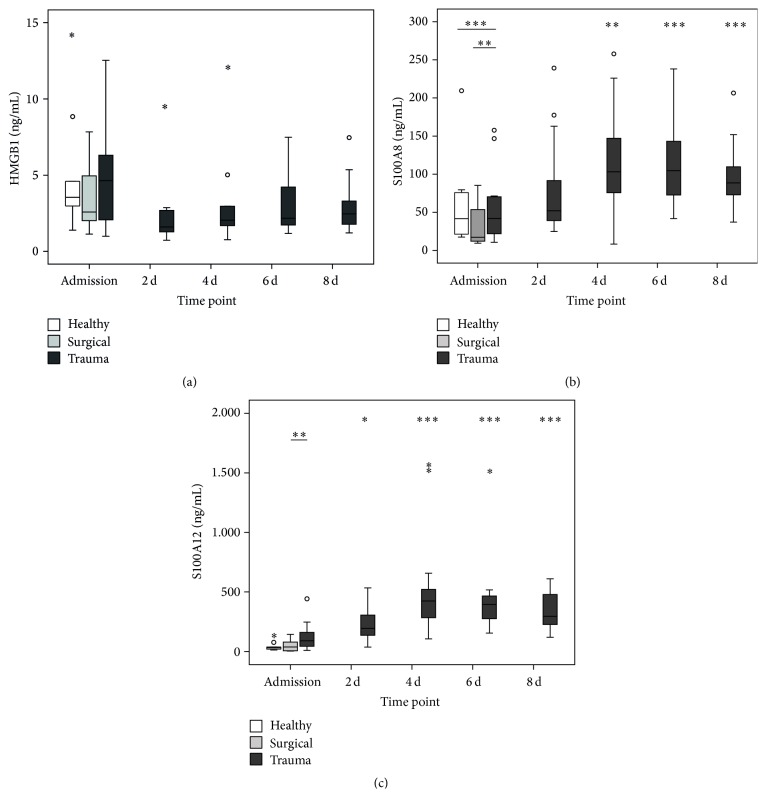
Plasma concentrations of proteinergic RAGE ligands after trauma. HMGB1 (a), S100A8 (b), and S100A12 (c) levels at admission (*n* = 16/10/10 for trauma/surgical/healthy) and 2 d (*n* = 16), 4 d (*n* = 15), 6 d (*n* = 11), and 8 d after trauma (*n* = 11). ^*∗*^ *P* < 0.05, ^*∗∗*^ *P* < 0.01, and ^*∗∗∗*^ *P* < 0.001 compared to trauma at admission (Mann-Whitney *U*).

**Figure 4:**
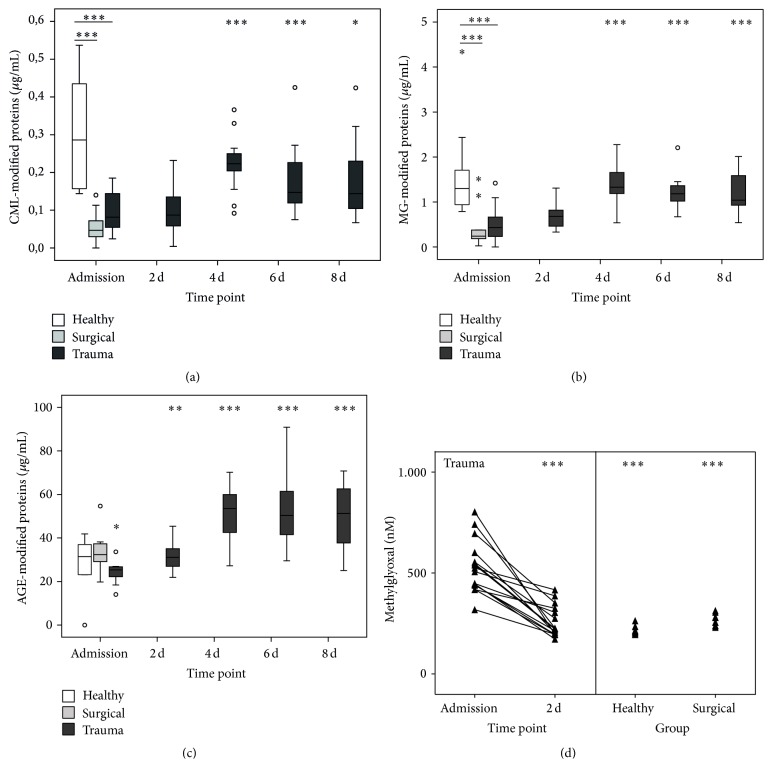
Plasma concentrations of metabolic RAGE ligands and methylglyoxal after trauma. CML- (a), MG- (b), and AGE-modified protein (c) and methylglyoxal (d) levels at admission (*n* = 16/10/10 for trauma/surgical/healthy) and 2 d (*n* = 16), 4 d (*n* = 15), 6 d (*n* = 11), and 8 d after trauma (*n* = 11). ^*∗*^ *P* < 0.05, ^*∗∗*^ *P* < 0.01, and ^*∗∗∗*^ *P* < 0.001 compared to trauma at admission (Mann-Whitney *U*).

A range of ligands from different origins and nature have been discovered for RAGE so far. We wanted to shed light on the question to which extent a panel of RAGE ligands actually occurs after trauma and might contribute to the pathophysiology. To our surprise, the plasma concentrations of the archetypical DAMP HMGB1 were changed neither immediately after trauma nor at any later time point (Figure 3(a)). In sharp contrast, S100A8 (Figure 3(b)) as well as S100A12 (Figure 3(a)) levels increased with a delayed kinetic and peaked at day 4 after trauma. The same kinetic was also found for AGE-modified proteins (Figure 4(c)). Taking a look at the other metabolic ligands, for example, CML- and MG-modified proteins, our study revealed a paradoxical kinetic with concentrations initially below healthy controls for surgical as well as trauma patients, recovering after 4 days back to normal (Figures 4(a) and 4(b)). Finally, we measured the concentration of the reactive carbonyl species methylglyoxal (MG) in the plasma, which is a relevant driver for the generation of AGEs (Figure 4(c)). In contrast to the decreased concentrations of CML- and MG-modified proteins, MG actually peaked very early exclusively in trauma patients and dropped back to normal values at day 2 after trauma. Overall, the results indicate an interwoven occurrence of early (IL-6, soluble RAGE, and methylglyoxal) and late (leucocytes, S100A8 + A12, and AGE-modified proteins) stakeholders of the immune response on the basis of an impaired immune function and low abundance of RAGE on monocytes in trauma patients. Moreover, the delayed generation of AGEs might be fueled by the high availability of glucose, which is a common feature of critical care patients.

## 4. Discussion

Our study is the first to our knowledge which examines the RAGE axis in patients after severe trauma in a longitudinal approach, including metabolic ligands and drivers, soluble receptor isoforms, and monocyte surface expression. Evidence for the importance of RAGE in this setting derives amongst others from polymorphism studies, presenting an association of infectious complications after trauma with a single nucleotide polymorphism (SNP) in the promotor region regulating RAGE expression [20]. Moreover, as a consequence of high basal expression levels of RAGE in the lung cells [21], ligation of the receptor might be causative to subsequent pulmonary dysfunction [22].

Earlier studies of our group and others show that soluble isoforms occur very rapidly and transiently after trauma and are associated, for example, with injury severity [16, 17]. These results are in line with the findings of our current study and are extended here in terms of control groups. In fact, patients after surgery do not show the drastic kinetic of RAGE occurrence like trauma patients, hinting at a molecular mechanism of generation which is rather depending on a deregulated homeostasis compared to the actual tissue injury, which is to the same extent present in the patients after major abdominal surgery. This idea is further supported by the fact that also patients with sepsis, a condition that represents the infectious counterpart to the sterile situation after trauma, exhibit high plasmatic levels of sRAGE [13, 14]. In the present study, plasma levels of IL-6 also showed a remarkable difference between patients after surgery and trauma, proving an early and fulminant SIRS as stated some decades ago [23]. Despite the differences in response between the groups, DAMPs have been proven to be important mediators of immune reactions after trauma and other conditions [6]. For HMGB1, an archetypical DAMP with high nuclear abundance in all somatic cells, the release after trauma has been reported earlier by Cohen and coworkers [24]. This is however conflicting with the results of our present study, where we cannot see any clear changes in HMGB1 levels. Nevertheless, HMGB1 has been reported earlier to induce a cross-tolerance to LPS [25] as well as a secondary state of “functional exhaustion” in mature monocytes [26]. Besides HMGB1, also a large arsenal of other DAMPs have been reported to be immunogenic drivers, not least our own mitochondrial DNA [27].

To estimate whether high levels of soluble RAGE are associated with a decreased surface expression on monocytes, flow cytometry experiments were performed. After an initial drop, the abundance of RAGE tended to increase in a portion of patients and then dropped again, while the proportion of RAGE^+^ monocytes stayed more or less the same. Although there was no significant difference, we cannot rule out that monocytes lose a part of their RAGE repertoire through, for example, enzymatic shedding by the metalloproteinase ADAM10 [28], what might be a counterregulatory mechanism to avoid excessive activation of inflammatory signaling cascades. In support of this idea, our patients show a persistent decrease of HLA-DR over the whole observation time, a marker that is a valid surrogate for monocytic immune function [29]. Nevertheless, a residual expression of RAGE on monocytes might still be sufficient for a basal signaling level, fueled by the delayed occurrence of immunogenic ligands of RAGE, namely, S100A8 and S100A12. The last one has also been reported to be capable of binding to and activating TLR4 in a proinflammatory fashion [30]. The same holds also true for S100A8, which has above all proven to induce a secondary state of hyporesponsiveness in monocytes [31], mimicking the clinical picture we observe in our cohort and similar to the well-known state of endotoxin tolerance [32]. In addition, we also found AGE-modified proteins to be increased in a delayed but profound manner. This might be an indicator of metabolically relevant changes in trauma patients, leading primarily to increased glucose levels and secondarily, in combination with the oxidative stress, to a generation of the aforementioned AGEs. One explanation might be the known presence of a transient, inflammation-induced insulin resistance [33], inducing diabetes-like metabolic conditions. Accordingly, “real” diabetes mellitus has been shown to be associated with an increased plasmatic AGE level and interestingly also a higher plasmatic methylglyoxal (MG) load [34], a feature we also observe in our trauma patients and we found out earlier in patients with sepsis [35]. MG is a member of reactive carbonyl species, which might influence cellular function in several ways. In the context of diabetes, MG has been shown to very distinctively modify and hyperactivate Na_v_1.8, a sodium ion channel expressed on nociceptive sensory fibers, resulting in the sensation of neuropathic pain [36]. Interestingly, Na_v_1.8 can also be activated by bacteria-derived formylated peptides, leading to pain but even more astonishingly to a modulation of the local immune reaction [37]. Combining these findings with our own results of high MG levels after trauma, two assumptions arise: first, there seems to be an intracellular catabolic change, leading to shunting of the energy flux from glycolysis to MG generation rather than to citrate cycle. Secondly, MG generation might be at the same time a proinflammatory stimulus as well as a trigger for an early mechanism to counteract excessive inflammation and thereby prevent harm to the organism.

Our pilot study implies some limitations regarding small sample size as well as the singular sampling of the surgical patients. In addition, trauma patients discharged from intensive or intermediate care unit were not followed up, what leads to a “survivorship bias.”

In summary, our study sheds light on all levels of the RAGE axis, from ligands over decoy receptors to surface RAGE expression. Intriguingly, we found a late occurrence of the proteinergic ligands S100A8 and S100A12 as well as high levels of AGE-modified proteins. In combination with the profound depression of immune responsiveness, which renders the patients vulnerable for an infectious encounter and the lack of IL-6 on later time points, one might hypothesize RAGE (and its ligands) to be contributing drivers to this refractory immune condition. As common in the immune system, the role of RAGE might be bivalent one, initially driving the sterile systemic immune reaction together with other receptors known to recognize DAMPs (e.g., TLR4) and secondarily a responsibility for the compensatory immune dysfunction of phagocytes (e.g., monocytes). However, as our study is based on the clinical setting, we are not able to unravel causal relationships. This task must be performed in a controlled setting, for example, cell culture in order to accurately pinpoint the role of RAGE in cellular function. If our very early and preliminary findings would hold true, one might speculate in trauma patients with sustained immune dysfunction about the rationale for a “paradoxical” treatment of disrupting the RAGE, ligand interaction by, for example, small molecule inhibitors (e.g., FPS-ZM1), which are currently tested in clinical trials for their potential use to prevent RAGE-mediated amyloid transport over the blood brain barrier [38]. The ultimate goal must be to balance out immunity in our patients to have as much response as needed, but without losing responsiveness in a compensatory fashion.

## Supplementary Material

Supplementary figure 1 contains figures of routine laboratory parameters of the trauma cohort over time, including leucocytes, C-reactive protein (CRP) and blood glucose.

## Figures and Tables

**Table 1:** Baseline characteristics of the three investigated groups.

	Trauma	Surgical	Healthy
Patients	16 (100)	10 (100)	10 (100)
Sex, male	14 (87.5)	8 (80)	6 (60)
Age, y	44.1 (19–69)	63 (48–76)	38 (28–55)
28-day mortality	2 (11)		
ISS	34.1 (19–51)		
Cause of injury			
Car accident	7 (43.75)		
Motorcycle accident	3 (18.75)		
Pedestrian accident	1 (6.25)		
Fall	2 (12.5)		
Unknown or other	3 (18.75)		
Injuries^#^			
Fracture of the extremities	9 (56.25)		
Fracture of the axial skeleton	10 (62.5)		
Abdominal trauma	12 (75)		
Thorax trauma	14 (87.5)		
Head injury	5 (31.25)		
Surgical procedure			
Esophagectomy		2 (20)	
Pancreatectomy		2 (20)	
Gastrectomy		3 (30)	
Hemihepatectomy		1 (10)	
Duodenectomy		1 (10)	
Hemicolectomy		1 (10)	

Values are given either as mean (range) for age and ISS or as *n* (% total); ^#^multiple naming possible.
