# Supplementary figures and images for: Role of the RAGE Axis during the Immune Response after Severe Trauma: A Prospective Pilot Study
Source: Mediators Inflamm. 2015 Dec 31;2015:691491. doi: 10.1155/2015/691491 (PMC4736010; doi:10.1155/2015/691491)

S1

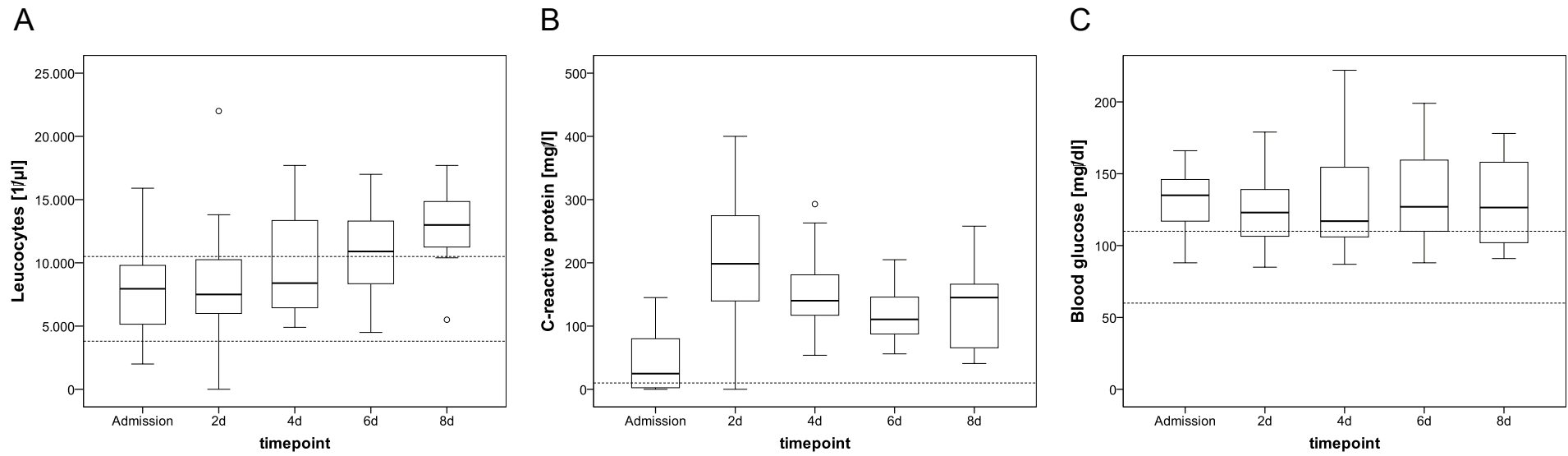

Supplement: Supplementary file 1 — Supplementary figure 1 contains figures of routine laboratory parameters of the trauma cohort over time, including leucocytes, C-reactive protein (CRP) and blood glucose. [file 691491.f1.pdf]
